# Morphological macrovascular alterations in complex regional pain syndrome type I demonstrated by increased intima-media thickness

**DOI:** 10.1186/1471-2377-13-14

**Published:** 2013-02-06

**Authors:** Nicola Derenthal, Tim Maecken, Elena Krumova, Alfried Germing, Christoph Maier

**Affiliations:** 1 Department of Pain Medicine, Berufsgenossenschaftliches Universitätsklinikum Bergmannsheil GmbH, Ruhr University Bochum, Bochum, Germany; 2 Department of Anaesthesiology, Intensive Care, Palliative Care and Pain Medicine Berufsgenossenschaftliches Universitätsklinikum Bergmannsheil GmbH, Ruhr University Bochum, Bochum, Germany; 3 Medical clinic II (Cardiology and Angiology), Berufsgenossenschaftliches Universitätsklinikum Bergmannsheil GmbH, Ruhr University Bochum, Bochum, Germany; 4 Present address: Department of Neurology, Berufsgenossenschaftliches Universitätsklinikum Bergmannsheil GmbH, Ruhr University Bochum, Bochum, Germany

**Keywords:** Complex regional pain syndrome, Macrovascular changes, Intima-media thickness, Inflammatory alterations

## Abstract

**Background:**

Although intima-media thickness (IMT) was increased in several inflammatory diseases, studies investigating whether the inflammatory processes lead to macrovascular alteration with increased IMT in complex regional pain syndrome (CRPS) lack.

**Methods:**

Using ultrasound (high-resolution B-mode), we compared bilaterally the IMT of the common carotid artery (CCA-IMT), the radial artery (RA-IMT), the brachial artery (BRA-IMT) and the quotient Q_RA/CCA_, in CRPS type I (n=17), peripheral nerve injury (PNI, n=17) and pain-free controls (PFC, n=22, matched to CRPS by gender, age and traditional cardiovascular risk factors). Statistics: Spearman’s correlation, paired t-test, ANOVA (p<0.05).

**Results:**

Compared to PFC, RA-IMT were significantly increased in both patient groups bilaterally (mean±standard deviation, CRPS affected side vs. PFC dominant side: 0.32±0.08 mm vs. 0.19±0.08 mm, p<0.001; PNI affected side vs. PFC dominant side: 0.27±0.09 mm vs. 0.19±0.08 mm, p< 0.05; CRPS non-affected side vs. PFC non-dominant side: 0.30±0.10 mm vs. 0.19±0.09 mm, p<0.001; PNI non-affected side vs. PFC non-dominant side: 0.25±0.10 mm vs. 0.19±0.09 mm, p<0.05) and Q_RA/CCA_ (CRPS affected-side vs. PFC dominant side: 0.49±0.12 vs. 0.30±0.11, p<0.001; PNI affected side vs. PFC dominant side: 0.41±0.10 vs. 0.30±0.11, p<0.05; CRPS non-affected side vs. PFC non-dominant side: 0.43±0.19 vs. 0.30±0.13, p<0.001; PNI non-affected side vs. PFC non-dominant side: 0.39±0.14 vs. 0.30±0.13, p<0.05), and BRA-IMT - only on the affected side in CRPS (CRPS: 0.42±0.06 mm vs. PFC: 0.35±0.08 mm; p<0.05). In CRPS, Q_RA/CCA_ was significantly higher on the affected side compared to PNI (p<0.05). However, only CRPS displayed within-group side-to-side differences with a significantly increased RA-IMT and Q_RA/CCA_ on the affected side (p<0.05). The CCA-IMT was comparable between all groups and sides.

**Conclusions:**

The increased IMT of peripheral arteries in CRPS suggests ongoing inflammatory process. Until now, only endothelial dysfunction has been reported. The presented morphological macrovascular alterations might explain the treatment resistance of some CRPS patients.

## Background

Complex regional pain syndrome type I (CRPS I) occurs as a severe complication after limb trauma without evidence of a peripheral nerve lesion. At the beginning of the disease patients present signs and symptoms like pain, edema, increased skin temperature and redness [1]. The underlying pathomechanisms of CRPS are still unclear, but the clinical signs and symptoms as well as other findings indicate the involvement of inflammatory processes for the development of CRPS [2]. For example, locally increased levels of pro-inflammatory cytokines like tumor necrosis factor alpha (TNF-α) and interleukin-6 (IL-6) were found in the affected limb of CRPS patients compared to the non-affected limb [3]. Furthermore, the systemic mRNA levels of some anti-inflammatory cytokines (interleukin-10) were decreased [4], whereas the systemic levels of pro-inflammatory neuropeptides including calcitonin gene-related peptide, bradikinin and substance P were increased [2]. Moreover, higher levels of endothelin-1, but low levels of nitric oxide have been described [5]. Furthermore, in CRPS patients with allodynia the concentration of plasma neuropeptid Y was lower on the painful side [6]. Additionally, lower concentration of plasma noradrenalin was found in CRPS patients with widespread allodynia as well as in those with hyperhidrosis [7].

Increased intima-media thickness (IMT), measured by high-resolution ultrasound, is a non-invasive marker of early arterial wall alteration. It can result from cardiovascular risk factors such as smoking, hypertension, diabetes mellitus and hypercholesterolemia. Anyway, it may not necessarily reflect pathologic changing because it is closely correlated to age [8]. However, an increased IMT of the common carotid artery (CCA-IMT) is directly associated with an increased risk of cardiovascular disease and its measurement is well accepted for clinical research [9].

Furthermore, there is a strong link between inflammatory markers and increased internal carotid IMT [10]. TNF-α and an increased CCA-IMT were not only positively associated in healthy middle-aged men [11] but also with inflammatory markers in different autoimmune diseases including rheumatoid arthritis [12], HIV-infected patients [13] or in patients with diabetes mellitus [14].

Modern high-resolution ultrasound systems can depict the macrovascular structure (and change) of intima-media thickness. To our knowledge, there are no studies on the IMT in CRPS, although, there are several reports of microvascular alterations in this disease, e.g. endothelial swelling and changes in the vessel luminal wall detected in a bone biopsy [15]. Furthermore, hypertrophic changes of the basal membrane layers of the capillaries were found in muscle tissue of CRPS patients, analyzed by light and electronic microscopy [16]. Additionally, in CRPS-affected skin the denerved vessels were hypertrophied with an increased size of lumen and thickened multi-laminated walls [17] and the number of migrated endothelial cells was increased [18].

The objective of the present prospective study was to investigate morphologic macrovascular alterations in patients with unilateral CRPS of the upper extremity by assessing the IMT of the radial, brachial and common carotid artery bilaterally in comparison to patients with unilateral peripheral nerve injury and matched pain-free controls using high-resolution ultrasonography.

## Methods

### Patients and pain-free controls

The study was approved by the local Ethics Committee of the Ruhr University Bochum, Germany (registration number: 3513–09). All subjects gave their written informed consent. 26 patients with the diagnosis CRPS type I of the upper extremity were recruited from the Pain Clinic of the University Hospital Bergmannsheil Bochum, Germany (February 2010 to September 2011). The diagnosis was made by two pain specialists (CM and EK) according to the clinical Budapest criteria (CRPS characteristics present in at least 3 of 4 symptom categories and at least 2 of 4 sign categories) [1]. All patients presented pain and dysfunction, which were disproportional to the inciting event, increased by movement of the affected limb and could not be explained by another disease. Additionally, all patients demonstrated the characteristic pattern of an enhanced bone metabolism in the late phase of a 99-m technetium-3-phase bone scintigraphy [19]. CRPS type II was excluded based on the absence of electromyographic and/or nerve conduction abnormalities. Furthermore, exclusion criteria were peripheral arterial obstructive disease stage IIb - IV by Fontaine, other morphological or functional vascular disease with impact on the affected extremity, advanced heart disease with angina pectoris stage III and IV according to the Canadian Cardiovascular Society, medication with direct vasodilatators, organic nitrate and molsidomin, sympatholytic drugs and vasoactive substances for circulation-promotion. The examination was aborted in nine of the 26 initially studied CRPS patients due to severe pain; most of them had longer lasting disease duration and were resistant to the common CRPS treatment, resulting in strong pain and limited hand function. Thus, finally 17 patients with unilateral CRPS I were compared to two control groups. Table 1 summarizes all relevant clinical data.

**Table 1:** Clinical data

**Variables**	**CRPS (n=17)**	**Patients with peripheral nerve injury (n=17)**	**Pain-free controls (n= 22)**
Gender (female, n (%))	8 (47%)	11 (65%)	12 (55%)
Age (years)	53 ± 14	53 ± 14	52 ± 13
Affected side (right), n (%)	11 (66%)	10 (60%)	
Diagnosis, n (%)	CRPS I, 17 (100%)	Neuralgia of median nerve, 6 (36%)	
		Neuralgia of ulnar nerve, 5 (30%)	
		Neuralgia of radial nerve, 5 (30%)	
		Neuralgia of median, ulnar, radial nerve, 1 (6%)	
Precipitating event, n (%)			
Fracture	6 (36%)	1 (6%)	
Crush injury	4 (24%)	8 (48%)	
Surgery	6 (36%)	4 (24%)	
Carpal tunnel syndrome	-	4 (24%)	
Other	1 (6%)	-	
Current analgesics, n°			
No medication	4 (24%)	7 (42%)	
Opioids	7 (42%)	6 (36%)	
Tricyclic Antidrepressants	3 (18%)	6 (36%)	
Anticonvulsants	6 (36%)	8 (48%)	
Coxibs	5 (30%)	2 (12%)	
Other non-opioid analgesics	9 (54%)	4 (24%)	
Cardiovascular risk factors, n (%)°			
Current smoker	4 (24%)	6 (36%)	5 (23%)
Past smoker	3 (18%)	3 (18%)	2 (9%)
Arterial hypertension	3 (18%)	5 (30%)	3 (14%)
Diabetes mellitus			
Typ I	-	2 (12%)	-
Typ II	1 (6%)	1 (6%)	1 (5%)
Hypercholesterolemia	1 (6%)	2 (12%)	1 (5%)
Patients with cardiovascular risk factors, n (%)			
None	6 (36%)	5 (30%)	11 (50%)
One factor	10 (60%)	6 (36%)	10 (45%)
Two factors	1 (6%)	5 (30%)	1 (5%)
Three factors	-	1 (6%)	-
Pain duration (months)	16 ± 13 (4…60)#	97 ± 12 (4…360)#	
Patients < 12 months pain duration, n (%)	10 (60%) #	1 (6%) #	
Current pain intensity (examination day, NRS 0–10)	4 ± 0	5 ± 4	
Maximal pain intensity (examination day, NRS 0–10)	6 ± 4	7 ± 4	
Patients with positive symptom categories, n (%) ° *			
Sensory	14 (82%)	13 (77%)	
Vasomotor	11 (65%)	8 (48%)	
Sudomotor/edema	14 (82%)	7 (42%)	
Motor/trophic	17 (100%)	15 (90%)	
Patients with positive sign categories, n (%) ° *			
Sensory	13 (78%)	12 (72%)	
Vasomotor	11 (66%)	7 (42%)	
Sudomotor/edema	14 (82%)	5 (30%)	
Motor/trophic	17 (100%)	15 (88%)	

Numeric values are in mean ± SD (range). Percentage values refer to the columns. # p<0.001 (Mann–Whitney U-test). CRPS: complex regional pain syndrome; NRS: numeric rating scala. ° Multiple answers possible. * According to the Budapest-criteria [1].

For the first control group 19 patients with PNI were recruited from the Pain Clinic of the University Hospital Bergmannsheil Bochum, Germany (February 2010 to September 2011). The diagnosis of unilateral peripheral nerve injury was made by experienced neurologists by history and extensive clinical and neurophysiological examination. Clinical examination included sensory abnormalities and motor deficits referring to the skin region and muscles innervated by the affected nerve. The diagnosis was approved by nerve conduction abnormalities and the present signs and symptoms were sufficiently explained by the nerve injury (Table 1). Electroneurographic findings were classified abnormal in case of a reduction of sensory or motor nerve conduction velocities, distal motor latencies, a prolongation of F-wave-latencies, a reduction or absence of the amplitude of compound motor action potentials after distal motor nerve stimulation or sensory nerve stimulation or if conduction blocks were present. Reference values are shown in Table 2. The exclusion criteria were the same as for the CRPS patients. Examination was aborted in two of the 19 initially studied PNI patients due to severe pain during the examination. Thus, 17 PNI patients were included and compared to 17 CRPS patients. In the two patient groups both affected as well as unaffected extremities were measured.

**Table 2:** In-house cut-off reference values of nerve conduction studies

	**Distal motor latencies (m/s)**	**Conduction velocity (m/s)**	**Amplitude (μV)**
**CMAP**			
Radial nerve	2.6	50.0	4
Median nerve	3.9	49.7	5.4
Ulnar nerve	3.3	50.6	4
**SNAP**			
Radial nerve		55.6	16
Median nerve		46.9	6.9
Ulnar nerve		44.6	5.8

Nerve conduction studies were classified as pathologic if the results were below these reference values. CMAP indicates compound muscle action potential; SNAP, sensory nerve action potential.

22 pain-free controls (PFC) served for a second control group. They were recruited from February 2010 until January 2011 among students, hospital staff or their relatives and were matched to the CRPS patients by gender, age (± five years) and common cardiovascular risk factors (cigarette smoking, diabetes mellitus, hypertension and hypercholesterolemia). Except from the matched cardiovascular risk factors all subjects were healthy without a history of trauma, neurological, vascular or inflammatory disease. After performing the Edinburgh inventory for assessment of handedness with the PFC group, the dominant hand was defined as test side (analogue to the affected side in the patients). Thus, the dominant side of the healthy subjects was compared to the affected side of both patient groups and the non-dominant side was compared to the non-affected side of both patient groups [20].

While performing the measurements the sonographer was not blinded to the clinical information.

### Investigations and measurements

To avoid inter-observer variability the study measurements were restricted to one examiner (ND) and were performed after a training phase with twenty other subjects with an experienced sonographer (TM). The correlation between the results obtained from TM and ND was analyzed with Spearman’s rank correlation coefficients (r=0.958, p<0.0001). A Bland-Altman-analysis showed no significant differences.

#### Intima-media thickness (IMT)

IMT measurements were performed on the subjects lying in supine position with the head in neutral position. The arms were in a slightly abducted, resting position with unclenched hands. A high-resolution ultrasound-system with a linear array was used (Sonosite Erlangen, Germany, HFLx38 13-6MHz). IMT-far-wall-measurements of the CCA were bilaterally performed according to the Mannheim-Consensus-protocol [21] using the SonoCalc-IMT-Software (version 4.1; long-axis-view of the CCA, one cm distal the bulb in a region free of plaques). The IMT is defined as the distance between the beginning of the tunica intima (far wall) and the beginning of the tunica adventitia. For reproducible measurements a high-quality image acquisition was used along a minimum length of 10 mm of an arterial segment as required [21]. The mean IMT was calculated by three consecutive examinations. IMT-far-wall-measurements of the brachial and radial artery were performed on both sides in a similar manner like those of the CCA (linear-probe set to highest resolution (Sonosite HFLx38 13-6MHz), SonoCalc-IMT-Software, multiple automatic measurements on 1 cm IMT). The BRA-IMT-measurements were performed while subjects were lying in supine position, the arm slightly abducted and rotated in resting position with unclenched hand (most comfortable position for study subject and investigator). Three consecutive examinations in long-axis-view of the BRA were performed bilaterally, whereas the linear array was positioned about 3–5 cm proximal the elbow. RA-IMT measurements were performed in the same manner. The transducer was positioned in the distal third of the forearm.

#### Quotient of the IMT of the radial and carotid artery (Q_RA/CCA_)

Increased CCA-IMT is directly associated with generalized atherosclerosis. To eliminate the impact of atherosclerotic diseases, we generated the Q_RA/CCA_ as a quotient (Q) of the IMT of the radial (RA) and common carotid artery (CCA):

(1) $$Q_{\mathrm{RA}/\text{CCA}}=\frac{\mathrm{RA}-\text{IMT}\;\left(\mathrm{mm}\right)}{\text{CCA}-\text{IMT}\;\left(\mathrm{mm}\right)}$$

### Statistical analysis

For statistical analysis the Statistica^®^-Software was used (StatSoft^®^ Inc., Tulsa, Oklahoma, USA). P-values <0.05 were considered statistically significant, p-values <0.01 highly significant. Descriptive statistics were performed for all variables. Mann–Whitney-U-test was performed to compare metric variables (e.g. age, duration of disease, pain intensity). Shapiro-Wilks W-Test was used to test normal distribution of the parameters. After confirming normal distribution for all IMT-values using the Shapiro-Wilks W-Test, t-test for paired samples was conducted to investigate body side differences within the groups. Group comparisons of each body side were analyzed using a one-way analysis of variance (ANOVA). Levene’s test was performed to assess the equality of variance between the different samples. The post-hoc test (LSD) was performed to detect significant group differences. The correlations between mean IMT and disease duration and pain intensity were analyzed with Spearman’s rank correlation coefficients.

## Results

### Clinical data

The gender proportion, the mean age and the number of cardiovascular risk factors were comparable between the three groups. The proportion of patients with affection of the right or left hand as well as the reported current and maximal pain intensity were also similar in both patient groups. CRPS patients had a significantly shorter duration of disease (p<0.001) compared to patients with PNI. The pain duration was less than 12 months in only one patient of the PNI group vs. ten of the CRPS patients (p<0.001, Table 1).

### Mean common carotid intima-media thickness

The mean CCA-IMT was similar in all groups and sides (CRPS affected vs. non-affected side: 0.69±0.19 mm vs. 0.67±0.18 mm, n.s.; PNI affected vs. non-affected side: 0.66±0.12 mm vs. 0.65±0.11 mm, n.s.; PFC dominant vs. non-dominant side: 0.64±0.12 mm vs. 0.63±0.11 mm, n.s.) (Table 3). An additional table file shows these data in more detail (see Additional file 1: Table S1).

**Table 3:** One-way ANOVA analyzing between group differences

	**Between groups effect**	**Post-hoc (group)**
CCA-IMT mean affected side (mm)	n.s.	
CCA-IMT mean non-affected side (mm)	n.s.	
BRA-IMT mean affected side (mm)	<0.05	°
BRA-IMT mean non-affected side (mm)	n.s.	
RA-IMT mean affected side (mm)	<0.01	#,+
RA-IMT mean non- affected side (mm)	<0.01	#,+
Q_RA/CCA_ affected side	<0.01	*, #, +
Q_RA/CCA_ non-affected side	<0.01	#, +

* CRPS vs. PNI, p<0.05; # CRPS vs. PFC, p<0.001; ° CRPS vs. PFC, p<0.05; + PNI vs. PFC, p<0.05. CRPS: patients with complex regional pain syndrome; PNI: patients with peripheral nerve injury; PFC: pain-free controls. CCA: common carotid artery; BRA: brachial artery; RA: radial artery; IMT: intima-media thickness; Q_RA/CCA_: Quotient of the IMT of the radial and carotid artery.

### Mean brachial intima-media thickness

**Figure 1:**
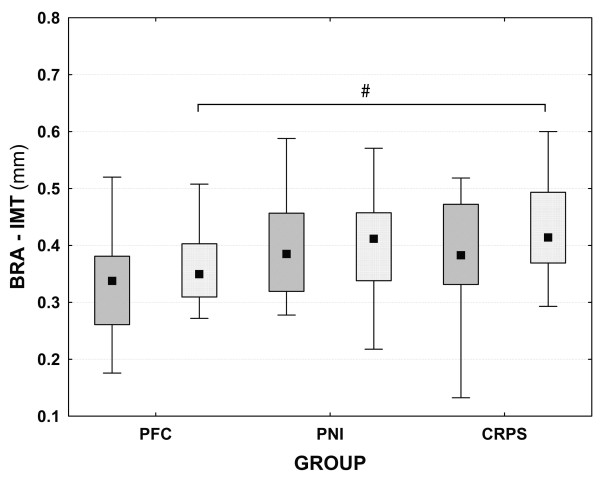
**Box-plot of the intima-media-thickness of the brachial artery (BRA-IMT); (mean ± standard deviation; 95% confidence interval). **# p<0.05 (ANOVA). PFC: pain-free controls; PNI: patients with peripheral nerve injury; CRPS: patients with complex regional pain syndrome. PFC group: dark grey boxes: non-dominant side, light grey boxes: dominant side; patients groups: dark grey boxes: non-affected side, light grey boxes: affected side.

Mean brachial IMT (BRA-IMT) was significantly increased on the affected side only in CRPS patients compared to PFC (0.42±0.06 mm vs. 0.35±0.08 mm, p: <0.05) (Table 3, Figure 1). There were no side differences in any of the groups (CRPS affected vs. non-affected side: 0.42±0.06 mm vs. 0.39±0.12 mm, n.s.; PNI affected vs. non-affected side: 0.40±0.08 mm v.s. 0.39±0.09 mm, n.s.; PFC dominant vs. non-dominant side: 0.35±0.08 mm vs. 0.33±0.09 mm, n.s.; Additional file 1: Table S1).

### Mean radial intima-media thickness

**Figure 2:**
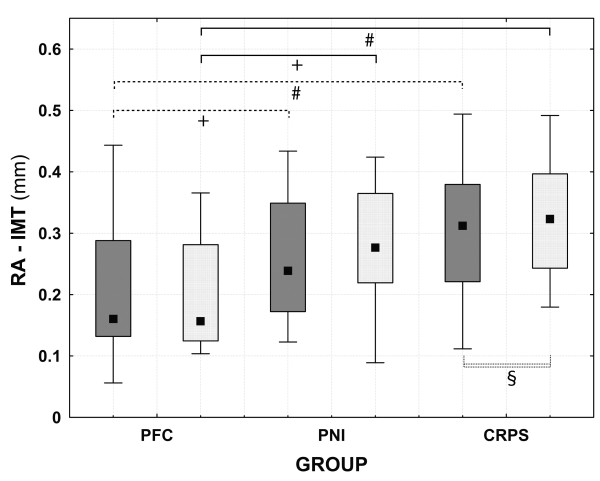
**Box-plot of the intima-media-thickness of the radial artery (RA-IMT); (mean ± standard deviation; 95% confidence interval). **+ p<0.05 (ANOVA); # p<0.001 (ANOVA); § p<0.05 (t-test for paired sample). PFC: pain-free controls; PNI: patients with peripheral nerve injury; CRPS: patients with complex regional pain syndrome. PFC group: dark grey boxes: non-dominant side, light grey boxes: dominant side; patients groups: dark grey boxes: non-affected side, light grey boxes: affected side.

The mean RA-IMT was significantly increased bilaterally in both patient groups in comparison to PFC without differences between the patient groups (CRPS affected side vs. PFC dominant side: 0.32±0.08 mm vs. 0.19±0.08 mm, p<0.001; PNI affected side vs. PFC dominant side: 0.27±0.09 mm vs. 0.19±0.08 mm, p< 0.05; CRPS non-affected side vs. PFC non-dominant side: 0.30±0.10 mm vs. 0.19±0.09 mm, p<0.001; PNI non-affected side vs. PFC non-dominant side: 0.25±0.10 mm vs. 0.19±0.09 mm, p<0.05) (Table 3, Figure 2). Only in CRPS patients the mean RA-IMT of the affected side was significantly increased compared to the non-affected side (p<0.05) (Figure 2, Additional file 1: Table S1).

### Quotient of the IMT of the radial and carotid artery (Q_RA/CCA_)

**Figure 3:**
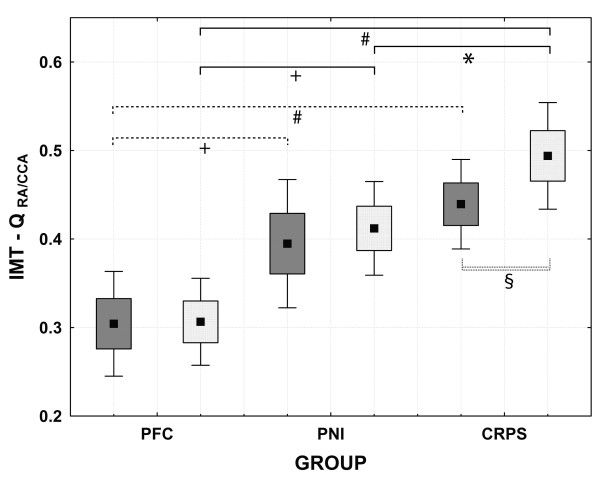
**Box-plot of the quotient of the intima-media thickness of the radial and carotid artery (Q**_**RA/CCA**_**); (mean ± standard deviation; 95% confidence interval).** + p<0.05 (ANOVA); # p<0.001 (ANOVA); * p<0.05 (ANOVA); § p<0.05 (t-test for paired sample). PFC: pain-free controls; PNI: patients with peripheral nerve injury; CRPS: patients with complex regional pain syndrome. PFC group: dark grey boxes: non-dominant side, light grey boxes: dominant side; patients groups: dark grey boxes: non-affected side, light grey boxes: affected side.

In CRPS patients the Q_RA/CCA_ was significantly higher on the affected side compared to the affected side in PNI patients (p<0.05) (Table 3, Figure 3). The Q_RA/CCA_ was significantly increased bilaterally in both CRPS and PNI compared to PFC (CRPS affected-side vs. PFC dominant side: 0.49±0.12 vs. 0.30±0.11, p<0.001; PNI affected side vs. PFC dominant side: 0.41±0.10 vs. 0.30±0.11, p<0.05; CRPS non-affected side vs. PFC non-dominant side: 0.43±0.19 vs. 0.30±0.13, p<0.001; PNI non-affected side vs. PFC non-dominant side: 0.39±0.14 vs. 0.30±0.13, p<0.05) (Table 3, Figure 3). Only in CRPS patients the Q_RA/CCA_ of the affected side was significantly increased compared to the non-affected side (p< 0.05) (Figure 3, Additional file 1: Table S1).

### Correlation between IMT and disease duration and pain intensity for both patient groups

CCA-IMT, BRA-IMT and RA-IMT did neither correlate with disease duration nor with pain intensity in the CRPS group and PNI group.

## Discussion

To our knowledge, this is the first study revealing morphological macrovascular alterations in CRPS patients measured by ultrasonography. The RA-IMT and the Q_RA/CCA_ were increased bilaterally compared to pain-free controls (PFC) both in CRPS and in PNI patients. The BRA-IMT of the affected side was increased only in CRPS patients compared to PFC. CRPS patients had a significantly higher Q_RA/CCA_ on the affected side compared to PNI patients. Only in CRPS the Q_RA/CCA_ and RA-IMT of the affected side were increased compared to the non-affected side. The CCA-IMT did not differ between all groups and sides.

Carotid IMT measurement by B-mode ultrasound is a common, reproducible and well accepted technique to evaluate the vascular status of patients [9]. In contrast to the carotid artery, peripheral IMT measurements have not been well established yet, probably due to difficulties in measuring the very small radial IMT-values (mean RA-IMT of 0.19±0.08 mm compared to 0.64±0.12 mm of the CCA-IMT in our pain-free controls). Unfortunately, the used software allowed no post-examination measurement of the intima-media-thickness. Therefore the blinding of assessment was impossible. However, Ku et al. demonstrated that RA-IMT ultrasound using frequencies of 12 MHz (13 MHz in the present study) is feasible and produced significant correlations to histological examinations [22]. There are only a few other studies of RA-IMT [22,23] or BRA-IMT[24] measurements that use ultrasound. Despite this limited experience, RA-IMT above 0.25 mm is regarded to be increased [22]. The mean RA-IMT of our PFC group was is in the same range of normotensive subjects as previously reported (0.19±0.02 mm) [23] and the mean RA-IMT of our patient groups were above or equal to 0.25 mm, indicating the validity of our RA-IMT results.

To eliminate the impact of atherosclerotic diseases we generated the Q_RA/CCA_ and matched our pain-free controls to CRPS patients on cardiovascular risk factors as well as age. Age for itself leads to an increase of 0.010 mm/y in the CCA both in males and females [25]. However, there were no significant differences in age between the groups in the present study; therefore, IMT differences between the groups cannot be solely explained by age effects.

Several studies have observed functional vascular abnormalities in CRPS [26], e.g. impaired endothelial function demonstrated using acetylcholine- and sodium nitroprusside-induced vasodilation combined with laser Doppler flowmetry [27]. In contrast, only a few authors described morphological abnormalities in the microvascular system. Our results of increased RA- and BRA-IMT are in line with the previously described microvascular alterations such as endothelial swelling and changes in the vessel luminal wall found in a human bone biopsy [15]; furthermore, extraordinary vascular hypertrophy characterized by severely thickened multi-laminated walls was found through immunohistochemical analysis of CRPS-affected skin [17] or an increased number of migrated endothelial cells [18].

Beside the vascular changes, there is also some evidence for inflammatory alterations in CRPS patients [3,4] and the important role of inflammatory processes for the development of this disease [2], including increased local concentration of inflammatory markers in the affected extremity of CRPS patients [3,4]. However, only a few studies give hints not only for local but also for systemic inflammatory processes in CRPS [28,29], such as increased levels of IL-8 and tumor-necrosis-factor receptor I/II in the affected arm of patients with acute CRPS I without significant differences compared to the non-affected arm [29]. Six years after disease onset the difference of TNF-α and IL-6-levels between the affected and the non-affected side was not significant anymore [28].

We hypothesize, that an increased IMT in CRPS patient is a sign of inflammatory processes. Unfortunately, we did not measure inflammatory mediators in the serum in our patients. However, we found a significantly increased radial IMT in the affected side compared to the non-affected side, in line with the previously reported high local concentration of inflammatory markers only in the affected extremity [3]. In accordance with these local findings we have observed unilaterally increased BRA-IMT only on the affected side. Similarly to the clinical signs and symptoms, which are presented mainly in the distal part of the affected limb, we have also observed a gradient in the macrovascular changes, which were more pronounced on the distal than on the proximal limb: The CCA-IMT was bilaterally not increased, while we found an unilateral moderate increase in the BRA-IMT and a more pronounced increase in the RA-IMT suggesting a local process in the distal limb. However, also the radial IMT of the non-affected side was significantly increased compared to PFC without having any signs or symptoms on that side, which could be in accordance with previous results indicating a systemic process. Merely in CRPS patients not only radial IMT but also brachial IMT was significantly increased in the affected side compared to PFC. However there was no significant difference between the affected and non-affected side, which is a further hint for a systemic process. However, the maximal IMT alteration in our study is located at the radial artery on the affected side. Even though our controls were matched to age, gender and risk factors, we found the highest Q_RA/CCA_ in CRPS patients indicating disease specific alterations. Thus, our findings suggest both a systemic and local inflammatory process. According to our hypothesis that inflammation might induce increased IMT in CRPS, several studies demonstrated the association between inflammatory markers such as TNF-α or IL-6 and an increased IMT of the CCA [10-12,14]. In contrast, to our knowledge there are no studies that examined the correlation between inflammatory signs and increased radial or brachial IMT.

Unexpectedly, we found an increased RA-IMT also in the PNI group compared to PFC, however without significant side differences in contrast to the CRPS group. In painful peripheral neuropathies significantly higher blood levels of IL-2 mRNA and TNF-α mRNA were measured compared to healthy subjects [30]. Furthermore, changes in plasma cytokines (IL-2, TNF) after nerve injury have been described [31]. To our knowledge, there are no studies which have observed intima-media thickening in patients with nerve injury. Although in our study the IMT of PNI patients was also increased, CRPS patients displayed the highest IMT. This was also supported by a significantly higher Q_RA/CCA_ of the affected side in CRPS patients compared to PNI patients.

It is important to notice that CRPS patients had significantly shorter mean disease duration than PNI patients. In only one patient of the PNI group vs. ten of the CRPS group the pain duration was less than 12 months. However, the disease duration in both groups was widely ranging (4–60 months in CRPS; 4–360 months in PNI), but neither CCA-IMT, nor BRA-IMT or RA-IMT correlated with disease duration in both patient groups. In other inflammatory diseases there was a positive correlation between disease duration and IMT [32]. However, there are hints that carotid intima-media thickening and radial wall hypertrophy are reversible [33]. We did not find a correlation between IMT and pain intensity probably because all our patients received a pain treatment and we did not included an untreated control group. Moreover, nine CRPS and two PNI patients were excluded from our study due to the impossibility to perform the ultrasound scan because of severe hyperalgesia and allodynia. Thus, these dropout-patients may result in an underestimation of our findings. However, assuming that the intima-media thickening results from inflammatory processes, our findings are in line with previous studies reporting absent correlation between inflammatory mediators and clinical course or outcome with high interindividual variation [34]. In our study it was impossible to prove the association between clinical signs and IMT because clinical signs were presented by nearly or more than 80% of the CRPS patients except from vasomotor signs (Table 1). However, patients with or without vasomotor signs did not show a difference in all mean IMT-values.

Our study was a cross-sectional analysis which served as a pilot project and which may not clarify completely the correlation of IMT-alterations and disease duration, pain intensity or clinical signs and symptoms by now. Even though the benefit of long-term measurement of carotid IMT progression to predict cardiovascular events is still under debate [35], longitudinal radial artery scan studies in CRPS within the first year after disease onset are necessary at the next step, having in mind the fast disease progression. Longitudinal studies are needed to analyze the time course of these alterations from the early to later stages of the disease as well as to examine whether an increased IMT of CRPS patients is reversible and, in case it is, if this reversibility is accompanied by clinical improvement. Repeated IMT-measurements may be used to test the effectiveness of CRPS-treatment. Future studies should also be combined with measurements of inflammatory mediators to prove a possible association between increased IMT and inflammatory markers. However, it should be considered that IMT measurements are limited by hyperalgesia and allodynia in CRPS patients as well as by the need for a physician with profound skills in sonography. In addition to that, our unexpected observation of locally enlarged IMT in PNI patients should be reevaluated in patients with different types of nerve injury with a greater sample size and with simultaneously measurements of inflammatory markers.

## Conclusion

In conclusion, we examined macrovascular alterations in CRPS patients using high-resolution sonography demonstrating for the first time bilaterally increased radial and unilaterally increased brachial IMTs. Further longitudinal studies are required to prove whether these alterations can be used as diagnostic or prognostic markers.

## Abbreviations

CRPS: Complex regional pain syndrome; IMT: Intima-media thickness; RA: Radial artery; CCA: Common carotid artery; BRA: Brachial artery; Q_RA/CCA_: Quotient of the IMT of the radial artery and common carotid artery; PFC: Pain-free controls; PNI: Peripheral nerve injury; TNF-α: Tumor necrosis factor alpha; IL-6: Interleukin-6; PNM: Patients with injury of the median nerve; PNRU: Patients with injury of the radial and ulnar nerve; y: Year; CMAP: Compound muscle action potential; SNAP: Sensory nerve action potential.

## Competing interests

The authors declare that they have no competing interests. This study was partly funded by the Ruhr University Bochum, FoRUM grant: F634-2008. TM has been involved in lecturing, education and training which was supported by the following companies: Sonosite (Nürnberg, Germany), GE Healthcare (Solingen, Germany), Esaote (Cologne, Germany) BK Medical (Pinneberg, Germany), Pajunk (Geisingen, Germany) and B. Braun (Melsungen, Germany). EK is a member of the IMI Europain project (see below). She is also supported by intramural fundings of the Ruhr University Bochum (FoRUM: grant number K046-10). CM is member of the IMI Europain project, including following industry members AstraZeneca, Pfizer, Esteve, UCB-Pharma, Sanofi Aventis, Grünenthal, Eli Lilly and Boehringer Ingelheim. As a member of the German Research Network on Neuropathic Pain, he has received research grants from the German Federal Ministry of Education and Research (BMBF, grants 01EM0107 & 01EM0502). He has received research grants from Pfizer, MSD, Mundipharma, Grünenthal, Astellas, and Lilly as well as consultant and/or speaker fees from Astellas, Sanofi Aventis, Wyeth, Pfizer, Mundipharma and Eli Lilly.

## Authors’ contributions

ND collected, analyzed and interpreted the data and wrote the first draft of the manuscript. TM educated ND in skills in sonography and supervised her during the study, analyzed the data, designed the figures and was involved in the preparation of the final report. EK was involved in patients’ recruitment, data interpretation and in the preparation of the final report. AG revised the paper. CM contributed to the acquisition of the funding, designed the study, supervised it during the data collection and was involved in the data interpretation and the preparation of the final report. All authors read and approved the final manuscript.

## Author’s information

This work is part of the doctoral thesis of Nicola Derenthal.

## Pre-publication history

The pre-publication history for this paper can be accessed here:

http://www.biomedcentral.com/1471-2377/13/14/prepub

## Supplementary Material

Additional file 1: Table S1 Intima-media thickness measured by ultrasonography in mm and quotient of the IMT of the radial and carotid artery (mean ± SD). Click here for file
